# Classic and Simultaneous Clinical Findings of an Exuberant Case of Netherton Syndrome: A Clinical Report

**DOI:** 10.5826/dpc.1104a65

**Published:** 2021-10-01

**Authors:** Ademar Schultz Junior, Karina Demoner de Abreu Sarmenghi, Ingrid Zon Sassine, Camila Pedruzze Machado, Priscila Pinto Barroso, Victor Catrinque Nascimento

**Affiliations:** 1 Dermatology Unit, Hospital Santa Casa de Misericórdia de Vitória, Vila Rubim, Vitória, ES, Brasil; 2 Escola Superior de Ciências da Santa Casa de Misericórdia de Vitória, Bela Vista, Vitória, ES, Brasil

**Keywords:** Netherton syndrome, dermoscopy, eyebrows, congenital ichthyosiform erythroderma

## Introduction

Netherton syndrome (NS) is a rare autosomal recessive genodermatosis, characterized by of ichthyosis linearis circumflexa (ILC), *bamboo hair* (trichorrhexis invaginata), and atopic dermatitis [1].

NS has no definitive cure. It is a rare dermatosis, which is difficult to diagnose, as the components of the syndrome may not be detected at the same time. It is therefore essential to deepen our understanding of this disease. In this article we present an exuberant case of NS with classic clinical findings, simultaneously to the diagnosis.

## Case Report

A 20-year-old dark-skinned woman attended her first dermatology visit due to erythema, flaking, and hair loss. At 3 months old, she had diffuse erythema and itchy scaling skin. At 3 years old, she had opaque and brittle hair, developing areas of alopecia in the temporal and occipital regions.

**Figure 1:**
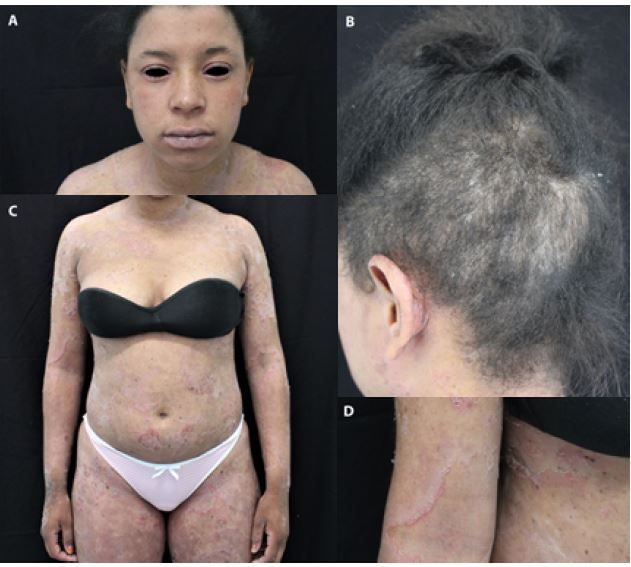
(A) An important xeroderma can be seen in the entire area of the face, extending to the neck and shoulders. There is erythema, edema and peeling of the eyelids, characterizing eczema. The lateral portion of the eyebrows stands out. (B) Alopecia in the occipital and temporal region. (C) Generalized involvement of the body by erythematous, scaly, and polycyclic lesions. (D) Erythematous, polycyclic, serpiginous, and annular lesion. It has a double scaling edge. This set characterizes the circumflex linear ichthyosis, which is a particular phenotype of the disease

Physical examination showed alopecia in the temporal and occipital regions, eyelid eczema, and eyelashes and distal third of eyebrows thinning (Figures 1, A and B). The patient had generalized erythroderma, lichenification of flexures and erythematous lesions with double-edge scaling, and serpiginous and polycyclic lesions at the level of the abdomen, upper, and lower limbs (Figures 1, C and D).

**Figure 2:**
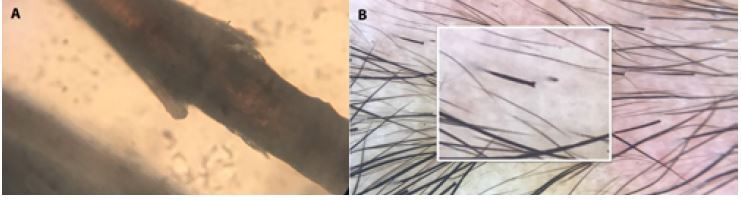
(A) Optical microscopy of the scalp hair (magnification ×40). Visualization of bamboo hair or invaginated trichorexis, a more specific alteration of the disease. (B) Detail of the trichoscopy of the right eyebrow revealing hair alteration “golf tee hair”.

At trichoscopy, *golf tee hair* was seen on the eyebrows and trichorrhexis invaginata at the optical microscopy of the hair (Figure 2, A and B). The patient maintained supportive treatment with skin hydration and a therapeutic plan to initiate systemic retinoids.

## Conclusion

NS comprises the alterations in the cornification of the skin due to mutations in the serine protease inhibitor Kazal type-5 (SPINK5), which encodes the serine protease inhibitor LEKTI (lympho-epithelial Kazal-type-related inhibitor) expressed on epithelial surfaces [1].

Diagnosis is difficult due to the multiple variables of the clinical presentation. Eventually, patients with NS manifest ichthyosis linearis circumflexa (ILC). The latter appears after 2 years of age, being characterized by erythematous-serpiginous lesions, which are polycyclic and show a double-edged scale. In some individuals with SPINK5 mutations, ILC is the only clinical manifestation of NS [1].

The basis for diagnosis is the trichorrhexis invaginata (TI) or *bamboo hair*, characterized by the invagination of the distal part over the proximal part of the hair shaft. However, it is not uncommon that hundreds of hair samples are examined for TI to be found. Thus, the diagnosis is often only established after several years of monitoring [1].

In some cases, there is a history of consanguineous parents, eosinophilia and increased IgE in peripheral blood. Atopy associated with NS can lead to an incorrect diagnosis of atopic dermatitis or severe eczema. Therefore, NS must be considered in children with atopic eczema who do not respond to standard treatment. Up to 75% of patients develop other atopic manifestations such as asthma, allergic rhinitis, angioedema, prutitus, and urticaria [1].

There is no specific treatment for NS. Throughout life, the treatment is symptomatic and individualized. Therapeutic options that can be used are phototherapy, systemic retinoids, and immunosuppressants. New studies are being carried out with anti-TNF treatment showing good perspectives. Recently, an exploratory study has shown good results with topic pimecrolimus 1% cream, revealing low systemic absorption and favorable safety and efficacy profile [2].

Concisely, skin lesions presenting simultaneously with hair changes are not widely common, which makes diagnosis difficult. Thus, we highlight the role of the dermatologist in the surmise of rare diseases based on the finding of specific skin and hair lesions, making it possible to perform an early diagnosis.
